# Effects of µ-Conotoxin GIIIB on the cellular activity of mouse skeletal musculoblast: combined transcriptome and proteome analysis

**DOI:** 10.1186/s12953-023-00221-w

**Published:** 2023-10-12

**Authors:** Han-xi Wu, Pei-min He, Rui Jia

**Affiliations:** College of Marine Ecology and Environment, Shanghai Ocean University, No.999, Huchenghuan Rd, Nanhui New City, Shanghai, 201306 P.R. China

**Keywords:** Conotoxins, Transcriptomics, Proteomics, µ-Conotoxin GIIIB

## Abstract

µ-Conotoxin GIIIB (µ-CTX GIIIB) is a polypeptide containing three disulfide bridges, produced by the sea snail Conus geographus. This study was aimed to explored the cytotoxic effects of µ-CTX GIIIB on mouse skeletal musculoblast (Sol8). Sol8 cells were exposed to ouabain and veratridine to establish the cell injury model, and then treated with µ-CTX GIIIB. CCK-8 was adopted to evaluate the cytotoxicity of µ-CTX GIIIB. Then, proteomics and transcriptome were conducted, and the explore the differentially expressed genes (DEGs) and differentially expressed proteins (DEPs) affected by µ-CTX GIIIB were found. Gene Ontology (GO) and Kyoto Encyclopedia of Genes and Genomes (KEGG) analysis was used to investigate the affected signaling pathways. µ-CTX GIIIB increased the cell survival rate of injured Sol8 cells. We found and identified 1,663 DEGs and 444 DEPs influenced by µ-CTX GIIIB. 106 pairs of correlated DEGs and DEPs were selected by combining transcriptome and proteome data. The results of KEGG and GO analysis showed that µ-CTX GIIB affected the cell cycle, apoptosis, DNA damage and repair, lipid metabolism and other biological processes of Sol8 cells. µ-CTX GIIIB could affected cell cycle regulation, DNA damage repair, and activation of tumor factors, with potential carcinogenic effects. Our results provide an important basis for the study of in vitro toxicity, the mechanism of toxicity and injury prevention by µ-CTX GIIIB.

**Supplementary Information:**

The online version contains supplementary material available at 10.1186/s12953-023-00221-w.

## Introduction

Conotoxins (CTX) are the natural peptide components extracted from the venom of conch snails (genus *Conus*). Most conch venoms are complex, containing at least 100 unique CTXs [1]. According to the consensus signal peptide sequence and cysteine framework, conotoxins can be divided into more than 20 superfamilies, such as A, M, O, P, S, T, I, V, Y, J, D, C and L [2]. Because CTXs have the characteristics of a wide variety, strong activity, easy synthesis and high selectivity, they are often used as a new source of drugs being developed for application in various areas, including pain relief, treatment of cancer, helping overcome smoking addiction and treating cranial nerve diseases [3].

The µ-conotoxins mainly act on various ion channels in organisms and belong to the M-superfamily of CTXs [4]. µ-Conotoxin GIIIB (µ-CTX GIIIB), isolated from the marine snail *Conus geographus*, is a polypeptide toxin containing 22 amino acid residues and three disulfide bridges [5]. It physically blocks the channel pore by binding to site I of the sodium channel, selectively blocking the voltage-gated sodium channel isoform of Nav1.4, which is predominantly expressed in muscle cells [6]. CTX is often used as a pharmacological research tool and its cellular effects deserve further study. Fang et al. found that ω-conotoxin (ω-CTX) has a neuroprotective effect on β-amyloid (Aβ)-induced neurotoxicity in Alzheimer’s disease (AD) cell models, increasing cell vitality and inhibiting inflammation and oxidative stress-related factors [7]. Salimi et al. extracted conotoxin directly from cone snails and found that the toxin was selectively toxic to chronic lymphocytic leukemia (CLL) cells and normal lymphocytes, and had a pro-apoptotic effect on CLL cells, but had no significant effect on normal cells [8]. Oroz-Parra et al. analyzed a peptide toxin (S-Cal14.1a) isolated from the sea snail *Conus californicus* that activates apoptosis in human lung cancer cell lines [9]. Several α-conotoxins are also widely used in anti-tumor studies [10–12]. In addition to being widely used as a specific sodium channel blocker, studies on µ-CTX GIIIB have mainly focused on organic synthesis, structure–activity relationship and the three-dimensional structure of amino acid residues [5, 6, 13], but there are few further studies on its toxicity.

Ouabain is a specific inhibitor of the ubiquitous Na+/K+-ATPase that is responsible for the active transport of Na + and K + across the plasma membrane of most animal cells [14]. Veratrine, a sodium channel activator, evokes presynaptic glutamate release though the opening of voltage-gated sodium channels [15]. In cells exposed to ouabain and veratrine (O/V), excessive opening of sodium channels occurs, leading to cell swelling and death. Intervention of O/V could be used to construct cell injury models [16]. In this study, we constructed a sol8 cell injury model by O/V intervention, and evaluated the cytotoxic effects of µ-CTX GIIIB in vitro. Transcriptome and proteome analysis were used to identify the differentially expressed genes (DEGs) and differentially expressed proteins (DEPs) in cells after µ-CTX GIIIB treatment. Based on the combined analysis of the omics data, the effects of conotoxin challenge on cell metabolism, signal transduction, cell cycle and other biological processes were preliminarily explored. The results provide reference for the study of in vitro toxicity and injury prevention of µ-CTX GIIIB.

## Materials and methods

### Cell culture

Sol8 cells (mouse skeletal muscle progenitor cells) (Otwo Biotech, China) were cultured in a 6-well cell culture plate with high glucose Dulbecco’s modified eagle’s medium (H-DMEM, Gibco, USA) containing 10% fetal bovine serum (FBS, Gibco, USA) and 5% penicillin-streptomycin solution. Cells were placed in an incubator (5% CO_2_, 37 ℃). After 48 h of cultivation, cells were passaged at a 1:6 ratio.

### Assessment of cytotoxicity and omics sample preparation (CCK-8)

Cell viability of Sol8 cells after exposure to ouabain (HY-B0542, MCE, USA) and veratrine (HY-N6691, MCE, USA) was evaluated by the CCK-8 assay. Experiment repeated independently 5 times. Ouabain (11018-89-6, 99.96%) and veratrine (71-62-5, 99.96%) were obtained from MedChemExpress (NJ, MCE), and dissolved in complete culture medium at a concentration of 10 µM and1 µM, respectively. Cells were seeded in 96-well plates (Thermo Fisher Scientific, USA) with a density of 2,000 cells/well, and cultured in 100 µL complete medium for 24 h. Subsequently, different doses of ouabain and veratridine solutions (0, 2, 4, 6, 8, and 10 µL) were added to each well, and then supplemented the complete medium to 100 µL. After 24 h of incubation, 10 µL CCK-8 (TIANGEN, China) was added to each well. The absorbance at 450 nm was measured using a microplate reader and cell viability was calculated. Finally, 5 µL ouabain and veratridine solution was selected to construct a Sol8 cell injury model according to the cell viability.

The effects of µ-CTX GIIIB on the Sol8 cell injury model were evaluated by CCK-8 assay. In brief, cells were seeded in 96-well plates (2,000 cells/well) for 24 h incubation. Blank control group, O/V group and experimental group were set up. The blank group was added with 100 µL complete medium. The O/V group and the experimental group were both added with 5 µL ouabain and veratridine solution, and the experimental groups were also supplemented with different doses of µ-CTX GIIIB (140678-12-2, Apeptide, China) solution (the final concentration was 0, 20, 40, 60, 80, 100, 200, 400, 600, 800 and 1000 nM). All wells were supplemented with medium to 100 ml. Subsequently, cell viability was calculated by CCK-8 assay.

### RNA extraction, library preparation and Illumina Hiseq xten sequencing

Cells were seeded in 6-well plates with a density of 2 × 10^5^ cells/well, and cultured for 24 h. When reached about 60% confluency, cells were exposed to varying levels of the µ-CTX GIIIB (0 and 1 µM) for 24 h. Sol8 cells treated with 1 µM µ-CTX GIIIB were named as the experimental group, and treated with 0 µM µ-CTX GIIIB were named as the control group. Three replicate wells were set up for each group. Then, cells were collected using a cell scraper and subjected to transcriptome sequencing.

TRIzol (Invitrogen, USA) was used to extract total RNA from Sol8 cells. Genomic DNA was removed using DNA enzyme I (TaKara Bio Inc., Shiga, Japan), and then the quality and purity of total RNA was evaluated by ND-2000 (NanoDrop, Wilmington, DE, USA). RNA-sequencing transcriptome libraries were prepared using 1 µg total RNA (OD260/280 = 1.8–2.2, OD260/230 ≥ 2.0, RIN ≥ 6.5, 28 S:18 S ≥ 1.0, > 1 µg) and a TruSeq™ RNA Sample Preparation Kit (Illumina Inc. San Diego, CA, USA). The mRNA was separated by polyA selection using Oligo (dT) beads and fragment buffers. Then, a SuperScript double-strand cDNA synthesis kit (Invitrogen, USA) and random hexamer primer (Illumina, USA) were used to construct double-stranded cDNA, and terminal repair phosphorylation and A-base addition were performed. The 300 bp cDNA fragment was amplified by PCR using Phusion DNA polymerase (NEB, Ipswich, MA, USA) and quantified by TBS380. A 300 bp cDNA target fragment was screened for Phusion DNA polymerase (NEB, USA) PCR amplification with 15 PCR cycles. After TBS380 was quantified, an Illumina HiSeq XTEN /NovaSeq 6000 sequencer (2 × 150 bp reading length) was used to sequence the RNA-Seq library at the pair end. Finally, the RNA-Seq library was sequenced using the Illumina HiSeq XTEN /NovaSeq 6000 sequencer (2 × 150 bp reading length).

### Read mapping

In order to control the quality, the original end readings were cut into pairs using SeqPrep (https://github.com/jstjohn/SeqPrep) and Sickle (https://github.com/najoshi/sickle). The directional patterns of reads were cleaned separately using HISAT2 (http://ccb.jhu.edu/software/hisat2/index.shtml) software and compared with the reference genome. Each example map was read by StringTie with a method based on a reference assembly.

### Identification of DEGs

In order to screen the DEGs between the two samples, the expression level of each transcript was calculated according to the transcripts per million reads (TPM) method. Gene abundance was calculated based on quantitative analysis using RSEM (http://deweylab.biostat.wisc.edu/rsem/). In essence, using the Q value 0.05 DESeq2 differential expression analysis, when |log2FC| > 1 and Q value ≤ 0.05 (DESeq2 or EdgeR) /Q value ≤ 0.001 (DEGseq), the gene was considered to be significantly differentially expressed. In addition, the Gene Ontology (GO) annotation analysis is carried out by Goatools(https://github.com/tanghaibao/Goatools) and the Kyoto Encyclopedia of Genes and Genomes (KEGG) function enrichment analysis is carried out by KOBAS (http://kobas.cbi.pku.edu.cn/home.do) [17].

### Quantitative real-time polymerase chain reaction (qRT-PCR)

To verify the accuracy of transcriptome data, seventeen genes were selected from the DEGs. And the remaining samples were used for qRT-PCR to verify the expression levels of nine genes. Table 1 shows the primers designed for the nine genes. cDNA was synthesized from RNA samples using HiScript Q-RT SuperMix and qRT-PCR was performed on an ABI7300 fluorescence quantitative PCR instrument (Bio-Rad, Hercules, CA, USA) and ChamQ SYBR Color qPCR (Vazyme Biotech, Nanjing, China). GAPDH was used as the reference gene for three replicates in each sample, and the relative fold changes of the expression levels of target genes in the samples were calculated by the 2^−∆∆CT^ method.

**Table 1:** The primer sequences used for qRT-PCR

Gene name	Sequence(5’-3’)
CDK2	F: CTCCCTGTTGGCACACTGAT
	R: ACTGGCTTGGTCACATCCTG
CCNB1	F: TGCCTGCAAATGCCTGGTTT
	R: AGTTACACCTTTGCCACAGC
CCNB2	F: CTCTTGCCCCTCAGTCATGT
	R: TTACATGACGGCACACACCG
TSP1	F: ACACCGAAAGGGACGATGAC
	R: TCTCTGTTCCAGGGCTTTGC
Plk1	F: CCGCAATTACATGAGCGAGC
	R: CGCTCACTCCACATTCAACC
BRCA1	F: GAGGAACGGGCTTGGAAGAA
	R: GGCCTTCTGGATTCTGGCTT
Taok2	F: CTTCCAACCTGTCCCCTTCC
	R: GTGAGGGGGCTGAGATAACG
Acot2	F: CTCTTGGATGTCGTGGAAGC
	R: GCACAGCGGGAAGTAAGG
Vegfa	F: TACTGCCGTCCGATTGAGA
	R: GCTGGCTTTGGTGAGGTTT
Tk1	F: ACAAGTGCCTGGTCATCAA
	R: GACTCCTGGGTCACATCG
Plin2	F: TGCCCTGCCCATCATCCA
	R: ACGCTGCCAGTCACTGCTCC
Hmgcs1	F: AGCCAAATTCTCAGCCCTAG
	R: TCCTCCTTGCCATACACG
Rrm2b	F: GACAGCAGAGGAGGTTGA
	R: TGAAAGCCATAGAAGCAG
Aurkb	F: TTCAACAGCCAGTCCACA
	R: CTTCTCCCGAGCCAAGTA
Ero1l	F: TTGGAGGATATGGAGTGT
	R: GCCTTCTAGCCAGTTGTA
Ctnnal1	F: GAGAAATGGGAAGATGAAG
	R: GGTGGCATAGAGGAATAAG
Kif20a	F: AAGGGCAGAACTGGCTCA
	R: CAGGTCAGGTGTCGGATG
GAPDH	F: CATCACTGCCACCCAGAAGACTG
	R: ATGCCAGTGAGCTTCCCGTTCAG

### Proteomic analysis

#### Total protein extraction and digestion

Sol8 cells were resuspended with lysis buffer [1% sodium dodecylsulfate acid (SDS), 8 M urea and protease inhibitor] for 30 min. Vortex mixing was performed every 5 min and ultrasonication was performed for 2 min (40 kHz, 40 w). After centrifugation (30 min, 4℃ 16,000 × g), the supernatant was removed and protein was quantified using a BCA protein assay kit (Pierce, Thermo Fisher Scientific, Waltham, MA, USA). 100 µL of lysate was added to every 100 µg of extracted protein at 37℃, followed by 10 mM TCEP buffer, and the protein was incubated for 60 min. Finally, iodine acetamide (40 mM) was added. The mixture was incubated in the dark for 40 min.

#### TMT labeling and high pH RPLC separation

The sample protein was precipitated in a 6-time volume of cold acetone for 4 h under − 20 °C conditions. Then the sample was centrifuged at 10,000 g at 4 °C for 20 min, and recovered by addition of 10,000 µL triethylammonium bicarbonate (TEAB) buffer (50 mM, Sigma, USA). Trypsin solution (1:50, Promega, USA) was added to each sample tube, and incubated overnight at 37 °C. Next, TMT reagent (Thermofisher, USA) in 50 µL acetonitrile was added and labeled for 2 h at room temperature. After 15 min with hydroxylamine at room temperature, sample desalination was performed, followed by drying in vacuo.

The mixed labeled samples were subjected to ACQUITY Ultra Performance Liquid chromatography (Waters Corporation, Milford, MA, USA) and ACQUITY UPLC BEH C18 column (1.7 m, 2.1 mm 150 mm; Waters) was used for separation. Briefly, the polypeptides were separated by gradient elution (Phase A: 2% acetonitrile, pH 10; Phase B: 80% acetonitrile, pH 10) over 48 min at a flow rate of 0.2mL/min. The peptides were eluted using the following gradient: 0 ~ 1.9 min, 0 ~ 0% B; 1.9 ~ 2 min, 0 ~ 5% B; 2 ~ 17 min, 5 ~ 5% B; 17 ~ 18 min, 5 ~ 10% B; 18 ~ 35.5 min, 10 ~ 30% B; 35.5 ~ 38 min, 30 ~ 36% B; 38 ~ 39 min, 36 ~ 42% B; 39 ~ 40 min, 42 ~ 100%B; 40 ~ 44 min, 100% B; 44 ~ 45 min, 100 ~ 0% B; 45 ~ 48 min, 0% B. Twenty fractions were collected from each sample and then pooled to produce ten total fractions per sample.

#### LC-MS/MS analysis

Labeled peptides were analyzed using a 9rkfsg2_ncs-3500r system (Thermo, USA) in on-line nanoflow liquid chromatography tandem mass spectrometry connected to a Q Exactive Plus quadrupole orbital well mass spectrometer (Thermo, USA) via a nanoelectro-spray ion source. The peptide mixture was added into a C18 reversed-phase column (75 μm × 25 cm; Thermo, USA), and the peptides were eluted at a flow rate of 0.3mL/min. The mobile phases consisted of buffer A (2% acetonitrile with 0.1% formic acid) and buffer B (80% acetonitrile with 0.1% formic acid). The gradient elution program was as follows: 0–4 min, 0–5% B; 4–66 min, 5 − 23% B; 66–80 min, 23 − 29% B; 80 − 89 min, 29 − 38% B; 89–91 min, 38–48% B; 91–92 min, 48–100% B; 92–105 min, 100% B; 105–106 min, 100–0% B. The Q Exactive mass spectrometer can automatically switch between MS and MS/MS acquisition, because it operates in data-dependent mode. The automatic gain control (AGC) target at 3e6 and the maximum fill time at 20 ms and full scan MS spectra (350–1300 m/z) were acquired at a resolution of 70,000.

#### Protein identification

The raw data files were analyzed using ProteomeDiscoverer (Thermo Scientific, Version 2.2) against the Mus_musculus database (http://asia.ensembl.org/Mus_musculus/Info/Index, Assembly Version GRCm38, 67856s). The MS/MS search criteria were as follows: The mass tolerance was 10 PPM Da 0.02 ms and MS/MS, trypsin 2 leakage was allowed, urea cysteine methylation and TMT N-terminal and lysine side chain polypeptides were fixed modifications, and methionine oxidation was a dynamic modification. The false discovery rate (FDR) of peptide identification was set as FDR ≤ 0.01.

### Statistical analyses

A total of 444 DEPs were obtained by the analysis and identification of proteomic data. Fold-change thresholds (> 1.2 or < 0.83) and *p* < 0.05 were applied to the screen for DEPs. We identified 121 upregulated and 323 downregulated proteins in the experimental group compared with the control group. All proteins identified were subjected to GO (http://www.blast2go.com/b2ghome; http://geneontology.org/) and KEGG pathway analysis (http://www.genome.jp/kegg/), and further analysis of the enrichment. Protein–protein interactions were analyzed using String V10.5.

### Parallel reaction monitoring (PRM) mass spectrometry analysis

PRM analysis was used to examine the DEPs obtained from the TMT experiment. Seventeen DEPs were randomly selected for PRM validation. In PRM analysis, the same method as TMT experiment was used for protein extraction, digestion and mass spectrometry analysis, and the obtained data were analyzed and integrated by Skyline software [18].

## Results

### µ-CTX GIIIB protects the Sol8 cells from cell injury induced by ouabain and veratrine

**Fig. 1:**
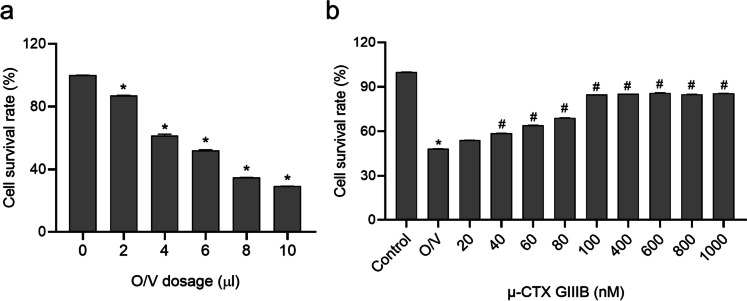
µ-CTX GIIIB protects the Sol8 cells from the cell injury induced by ouabain and veratrine (O/V). **a** The effects of ouabain and veratrine on cell survival rate of Sol8 cells was detected by CCK-8. *P < 0.05 vs. 0 group; (**b**) The effects of µ-CTX GIIIB on cell survival rate was detected by CCK-8. *P < 0.05 vs. Control group, ^#^*P* < 0.05 vs. O/V group

The main cellular effect of µ-CTX GIIIB is to block the ion channel (Nav1.4), which may affect the normal biological function of cells, but does not directly cause cell death. According to the in vitro bio toxicity detection method of paralytic shellfish toxin [19, 20], the cell injury model Sol8 (Nav1.4 channel protein was widely expressed) was established by ouabain (10 mM) and veratrine (1 mM), and then conotoxin of different concentration gradient was added to block sodium channel and protect the cells. We evaluated the cytotoxic effect of µ-CTX GIIIB by CCK-8 assay. First, we examined the cytotoxicity of O/V on Sol8 cells and found that the cell survival rate was decreased significantly with the increase of the dosage (Fig. 1a). Subsequently, 5 µL O/V was selected to establish the Sol8 cell injury model, followed by the addition of different concentrations of µ-CTX GIIIB. The CCK-8 results showed that µ-CTX GIIIB intervention increased the cell survival rate in O/V injury cells (Fig. 1b). These results indicated that the µ-CTX GIIIB protected Sol8 cells from O/V damage.

### Transcriptome and proteome analysis

**Fig. 2:**
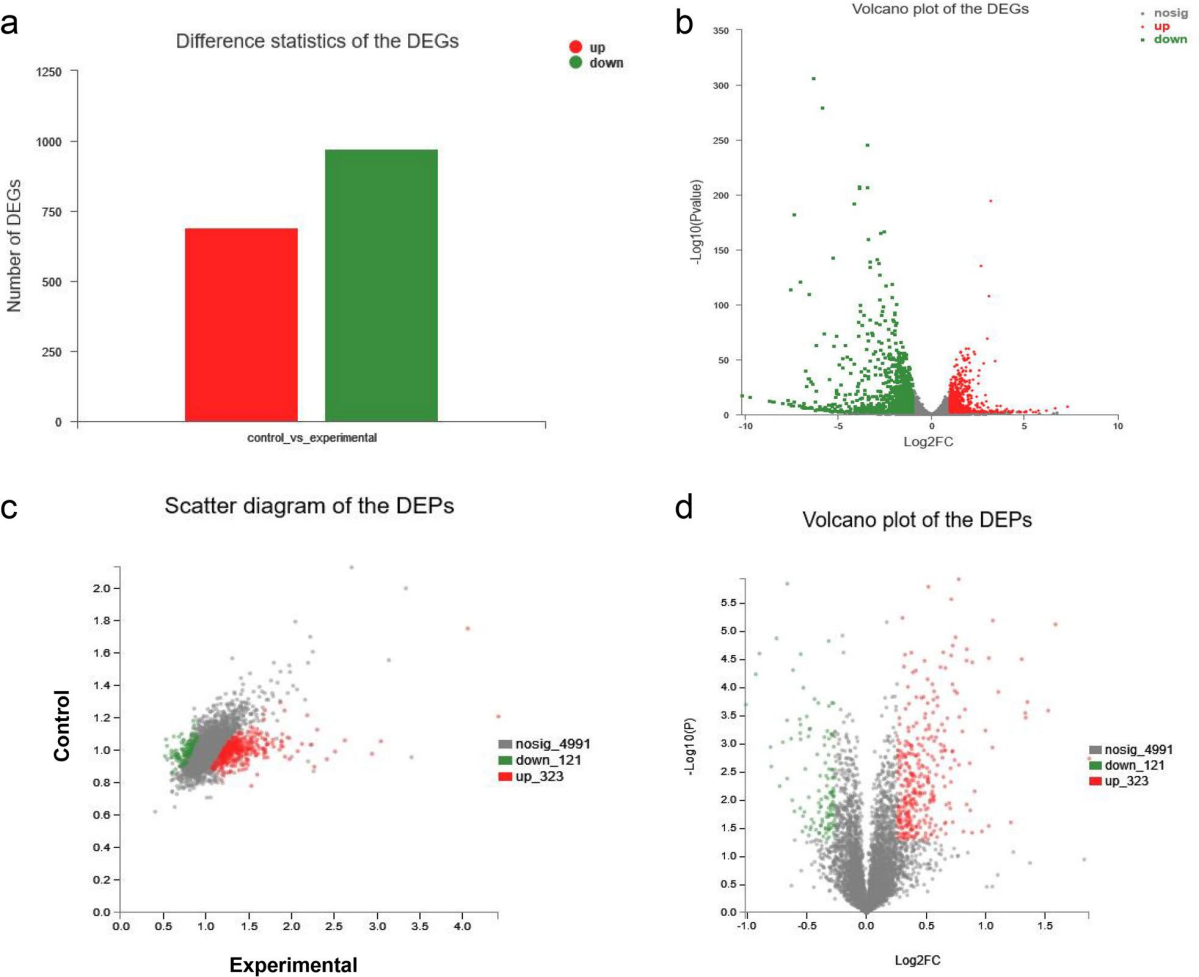
The DEGs and DEPs were found and identified by transcriptome and proteome analysis. **a** The Difference statistics of the DEGs. Red indicates upregulated genes and green indicates downregulated genes. **b** The Volcano plot of the DEGs. Compared with the control group, the X-axis represents the fold change between groups, and the Y-axis represents the *p* value difference of DEGs. The gray dots represent genes with no significant change, the red dots represent genes that are up-regulated, and the green dots represent genes that are down-regulated. **c** Scatter diagram of the DEPs, in which the x-axis and y-axis respectively represent the protein expression levels in two samples, and each point represents a specific protein. The closer a point is to 0, the lower the expression level is. The farther a point is to 0, the higher the expression level is. The gray dots represent proteins with no significant change, the red dots represent proteins that are up-regulated, and the green dots represent proteins that are down-regulated. **d** Volcano plot of the DEPs. The X-axis represents the fold change between two groups, and the Y-axis represents the *p* value difference of DEPs. The gray dots represent proteins with no significant change, the red dots represent proteins that are up-regulated, and the green dots represent proteins that are down-regulated

**Fig. 3:**
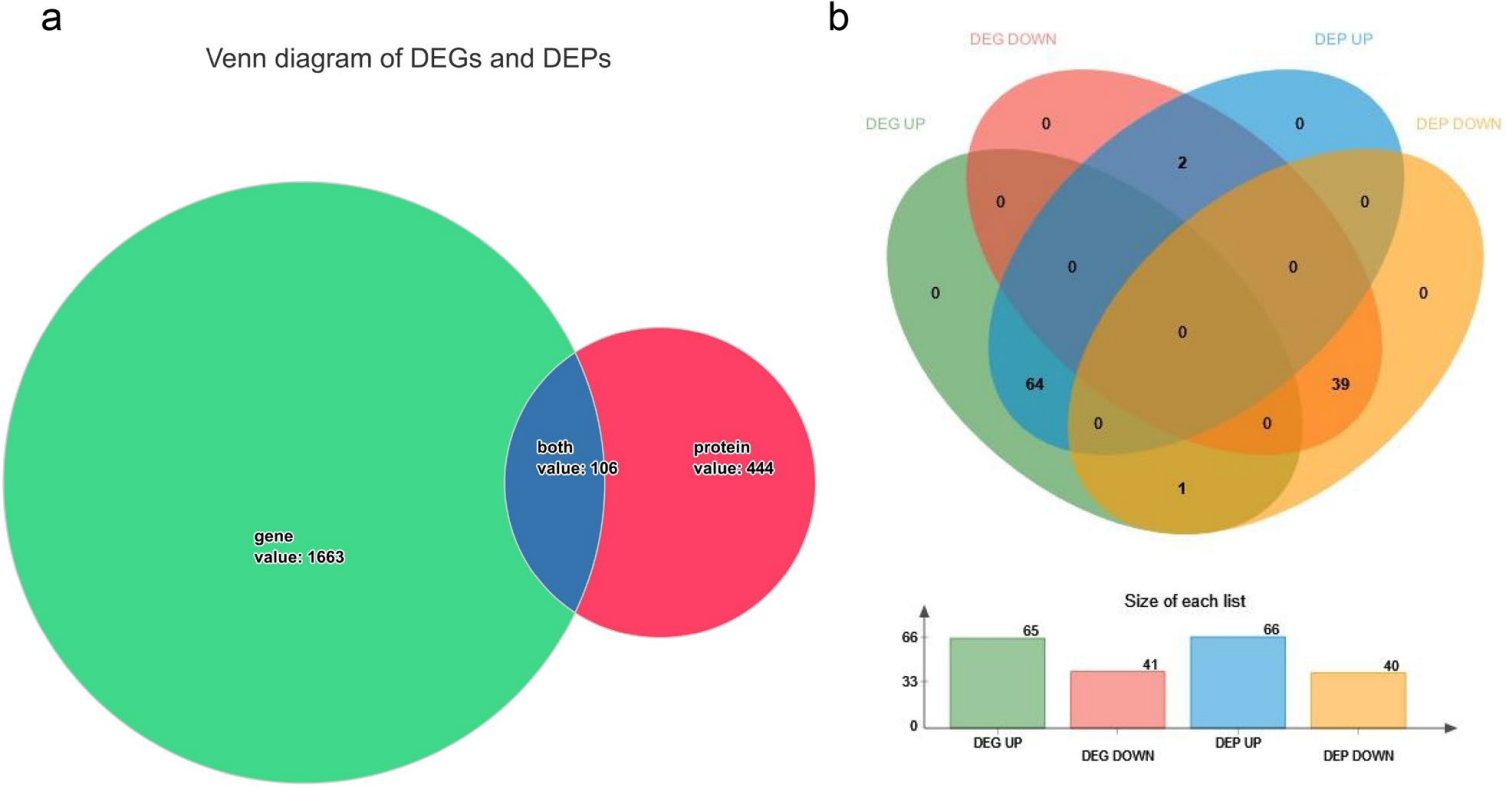
The comprehensive analysis of transcriptome and proteome. **a** Venn diagram of DEGs and DEPs. The green part represents DEGs, red represents DEPs, and blue represents associated genes and proteins. **b** Associated data Venn diagram. The diagram contains the up-regulated and down-regulated genes and proteins in associated data

In order to investigate the effects of high concentrations µ-CTX GIIIB (1µM) on the biological processes of cells, transcriptomic and proteomic analyses were performed. When the experimental group was compared with the control group, genes with a fold change > 2 or < 0. 5 and a *p*-value < 0.01 were considered to be differentially expressed. We identified 1,663 DEGs, including 691 up-regulated and 972 down-regulated genes (Fig. 2a, b and Table S1). For proteomic analyses, only proteins with a fold change > 1. 2 or < 0. 83 and *p* < 0.05 were considered to be differentially expressed. Analysis of the proteomic data revealed 444 DEPs, of which 323 were up-regulated and 121 were down-regulated (Fig. 2c, d and Table S2). Next, we comprehensively analyzed the transcriptomic and proteomic results. One hundred six protein/gene pairs were found in both the proteome and the transcriptome, but only 103 protein/gene pairs were down-regulated or up-regulated at the same time, among which 64 were up-regulated and 39 down-regulated (Fig. 3a, b and Table S3).

### GO and KEGG analysis

**Fig. 4:**
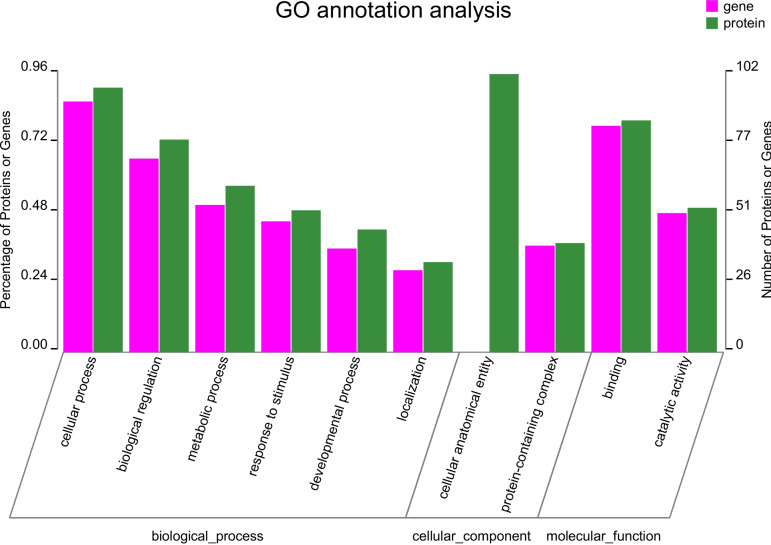
GO annotation analysis of DEGs and DEPs. Proteins and genes were divided into three major categories: biological processes, cellular components, and molecular functions. Green bars represent proteins and purple bars represent genes. The Y-axis (left) indicates the percentage of proteins or genes contained in the secondary classification to the total number of proteins or genes in the secondary classification, and the Y-axis (right) indicates the number of proteins or genes annotated to the secondary classification

The GO and KEGG analysis were performed to explore the biological functions of differentially expressed genes and proteins. According to GO functional analyses, the biological processes (BP), cellular components (CC) and molecular functions (MF) of DEGs and DEPs were investigated. In BP, proteins and genes were mainly associated with cellular processes, biological regulation, and metabolic processes. In CC, proteins and genes were categorized into protein-containing complex. In terms of MF, catalytic activity and binding were enriched (Fig. 4).

**Fig. 5:**
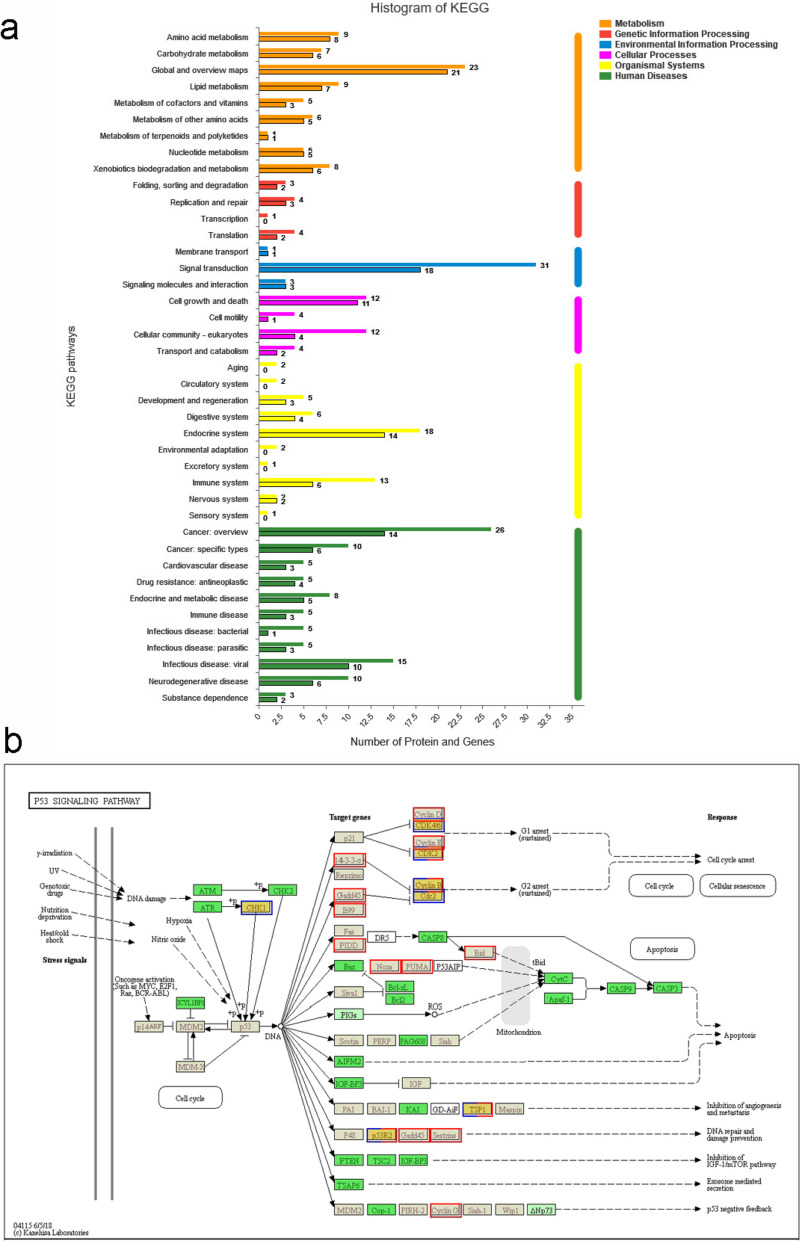
KEGG pathway analysis of DEGs and DEPs. **a** The Signal pathways affected by DEGs and DEPs. The borderless bars represent proteins and the borderless bars represent genes. The x axis indicates the number of genes and proteins that are matched, and the y-axis shows pathways in the KEGG classification. **b** The p53 signaling pathway influenced by DEGs and DEPs. In the figure, all green background boxes represent matched proteins of the candidate species, gray background boxes represent matched genes, and orange background boxes represent matched genes or proteins at the same time. The blue border represents the protein set and the red border represents the gene set. The red and blue border indicates the set of genes and proteins that are matched simultaneously

Based on KEGG analysis, 103 pairs of DEPs and related DEGs were assigned to 206 signaling pathways and categorized into six KEGG pathways (Fig. 5a and Table S4). The DEPs and DEGs were mainly enriched in metabolic pathways, aminoacyl-tRNA biosynthesis, p53 signaling pathway, microRNAs in cancer, PPAR signaling pathway, MAPK signaling pathway, PI3K-Akt signaling pathway and cell cycle. The p53 signaling pathway is the most abundant protein and gene pathway associated with cancer. We analyzed the effect of DEPs and DEGs on the p53 signaling pathway (Fig. 5b).

### qRT-PCR validation of DEGs

**Fig. 6:**
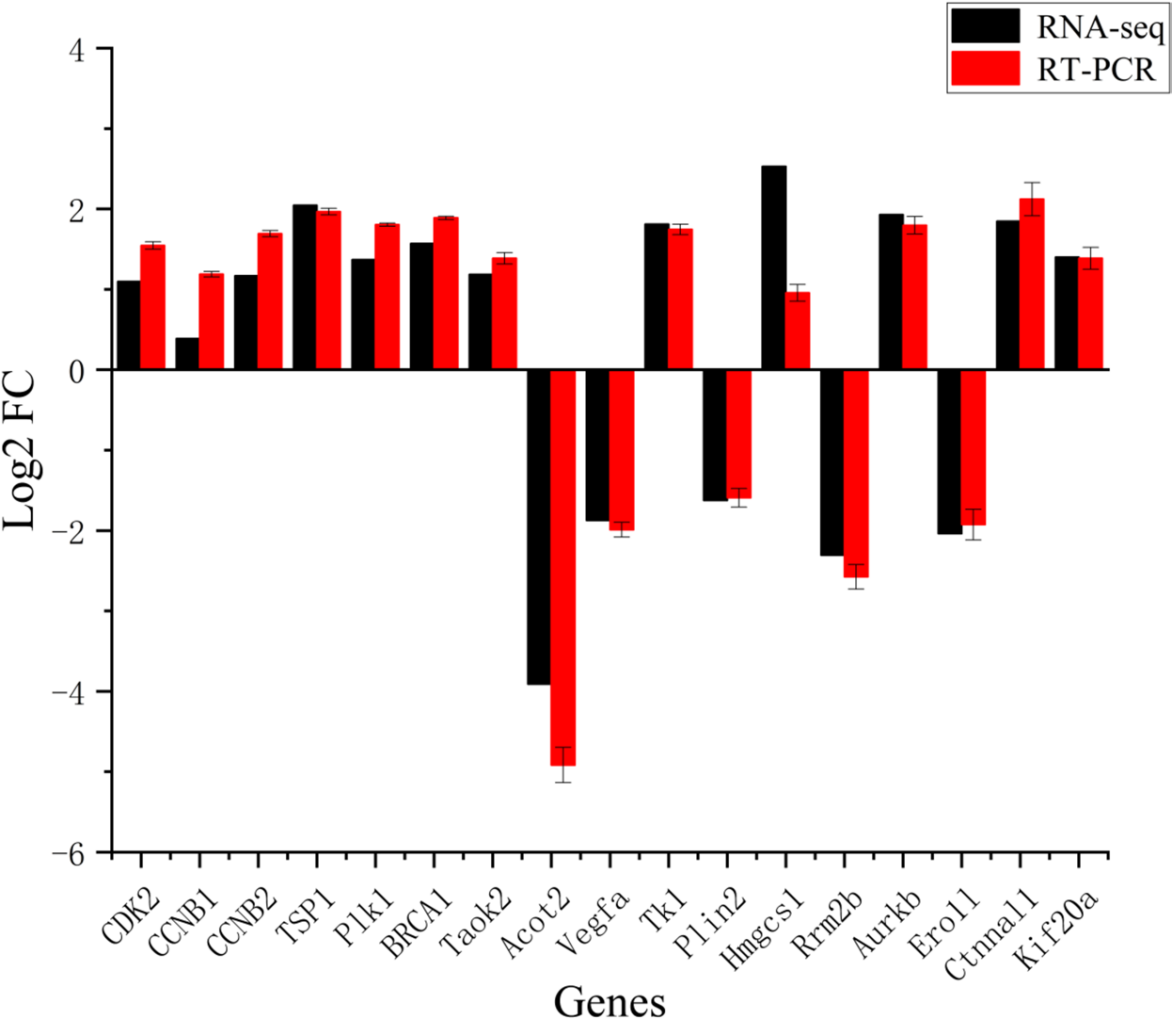
qRT-PCR validation of nine DEGs. The mRNA expression of CDK2, CCNB1, CCNB2, TSP1, Plk1, BRCA1, Taok2, Acot2, Vegfa, Tk1, Plin2, Hmgcs1, Rrm2b, Aurkb, Ero1l, Ctnnal1, and Kif20a were detected by qRT-PCR. Red bars represent the data of qRT-PCR and black bars represent the RNAseq data. Cyclin dependent kinase 2 (CDK2); cyclin B1 (CCNB1); cyclin B2 (CCNB2); thrombospondin 1 (TSP1); polo like kinase 1 (Plk1); breast cancer type 1 susceptibility protein (BRCA1); TAO kinase 2 (Taok2); acyl-CoA thioesterase 2 (Acot2); vascular endothelial growth factor A (Vegfa); thymidine kinase 1 (Tk1); perilipin 2 (Plin2); 3-hydroxy-3-methylglutaryl -coenzyme A synthase 1 (Hmgcs1); ribonucleotide reductase M2 B (Rrm2b); aurkb aurora kinase B (Aurkb); endoplasmic reticulum oxidoreductase 1 alpha (Ero1l); catenin (cadherin associated protein)alpha-like 1 (Ctnnal1); kinesin family member 20 A (Kif20a)

To verify the accuracy of transcriptomic data, seventeen genes were selected from the DEGs, and performed qRT-PCR validation. Experimental results showed that the trends in the expression expression of the seventeen genes were similar to RNAseq results, indicating the reliability of transcriptome data (Fig. 6).

### PRM data validation of randomly-selected proteins

**Fig. 7:**
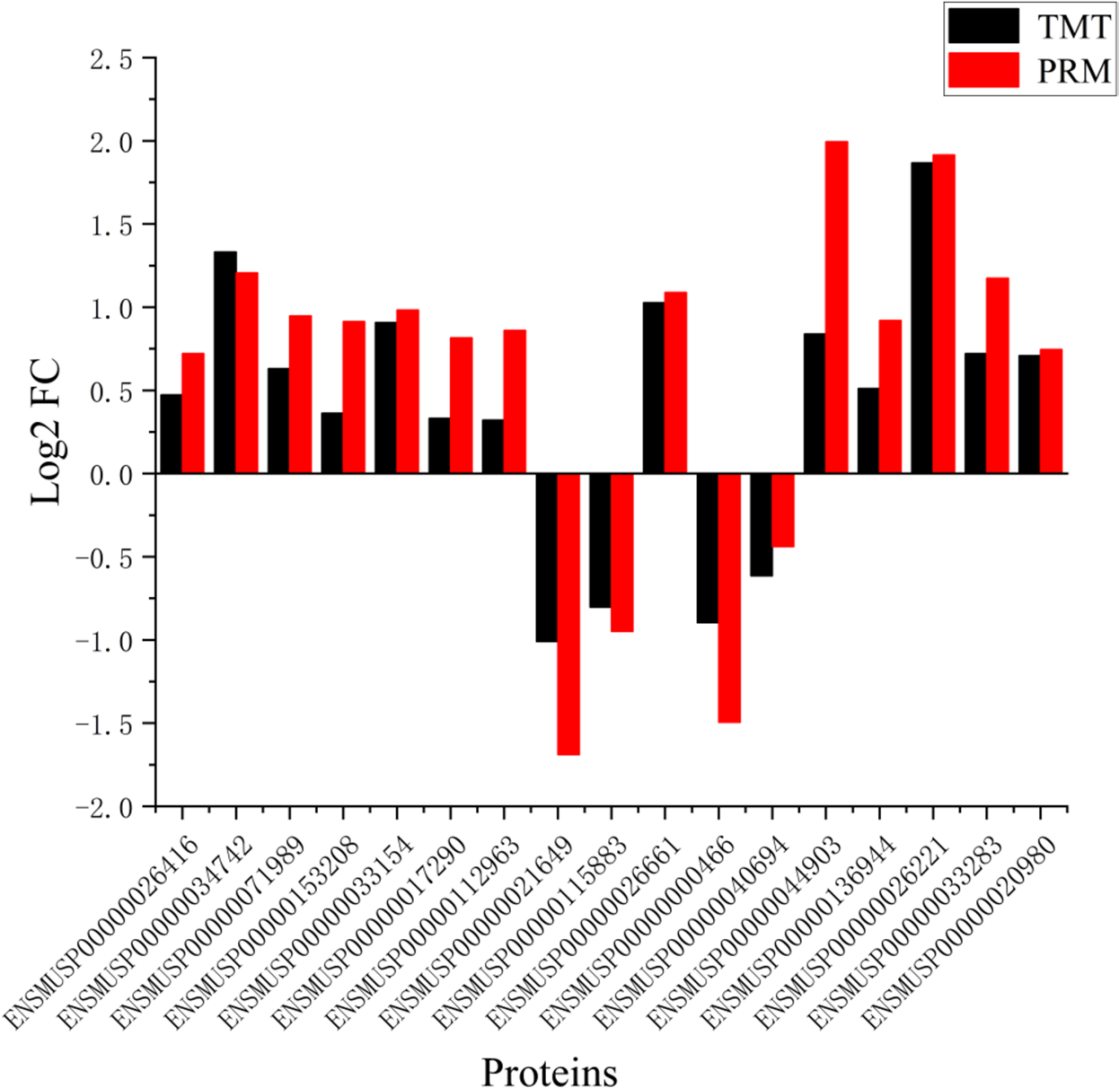
Validation of DEPs by PRM. The protein expression was detected by PRM analysis. Red bars represent the data of PRM and black bars represent the TMT data. ENSMUSP00000026416 (CDK2); ENSMUSP00000034742 (CCNB1); ENSMUSP00000071989 (CCNB2); ENSMUSP00000153208 (TSP1); ENSMUSP00000033154 (PLK1); ENSMUSP00000017290 (BRCA1); ENSMUSP00000112963 (Taok2); ENSMUSP00000021649 (Acot2); ENSMUSP00000115883 (Vegfa); ENSMUSP00000026661 (Tk1); ENSMUSP00000000466 (perilipin 2); ENSMUSP00000040694 (recombination signal binding protein for immunoglobulin kappa J region); ENSMUSP00000044903 (thrombospondin 1); ENSMUSP00000136944 (3-hydroxy-3-methylglutaryl-Coenzyme A synthase 1); ENSMUSP00000026221 (stearoyl-Coenzyme A desaturase 2); ENSMUSP00000033283 (ribonucleotide reductase M1); ENSMUSP00000020980 (ribonucleotide reductase M2)

In order to further verify the accuracy of proteomic data, seventeen proteins were selected from the DEPs screened by tandem mass tag (TMT) analysis, and the expression patterns of proteins in the samples were verified by parallel reaction monitoring (PRM). PRM analysis results were similar to TMT analysis results, which proved the accuracy of proteomic data (Fig. 7).

## Discussion

µ-CTX GIIIB is a polypeptide containing three disulfide bridges, which are widely used as a specific sodium channel blocker. Herein, we explored the effects of µ-CTX GIIIB on cell cytotoxicity by transcriptome and proteomics, and explored possible mechanisms using GO and KEGG analysis.

Our study found that µ-CTX GIIIB affected cell growth and death. The main enriched pathways in cell growth and death are the p53 signaling pathway, cell cycle and cellular senescence. In this research, combined transcriptomic and proteomic analysis revealed that most protein/gene pairs were simultaneously upregulated, including cyclin-dependent kinase 2 (CDK2), cyclin-B1 (CCNB1), cyclin-B2 (CCNB2), thrombospondin 1 (TSP1) and Polo-like kinases (PLKs). Overexpression of cyclin-associated proteins has been found in many cancers, and it is speculated that this may be the reason why conotoxin may promote the development of tumor cells. CDK2 drives cells through the G1/S transition by binding to E-cyclins, and causes cells to enter the S phase by interacting with A-cyclins [21]. CDK2 is a positive regulator of cell proliferation, and cell differentiation depends on its decreased activity [22]. In eukaryotic cells, CCNB1 and CCNB2 are cyclins which regulate the cell cycle and are closely related functionally [23]. Overexpression of CCB1 effectively induces cell division in post-mitotic mouse, rat and human cardiomyocytes [24]. Cyclin B1 and cyclin-dependent kinase 1 (CDK1) constitute the M phase promotors of mammalian oocyte meiosis. CDK1 can be activated by up-regulated Cyclin-B2 in the absence of CCNB1, indicating a potential compensatory mechanism between them [25]. In this study, cyclin and cyclin-dependent protein kinase were significantly upregulated, suggesting that µ-CTX GIIIB may affect the normal cell cycle and promote cell proliferation. TSP-1 is a stromal cell glycoprotein that significantly affects extracellular matrix structure and cell phenotype, which is mainly associated with the reconstruction of tumors and angiogenesis [26]. In addition, TSP-1 has different effects on cell proliferation and migration in different cells, having a promotional effect on smooth muscle cells but an inhibitory effect on endothelial cells [27]. The effect of toxin treatment on the expression of interstitial glycoprotein indicated that it could affect not only cell proliferation, but also intercellular adhesion and migration. PLKs are a class of potent regulators of the M phase with unique roles in spindle pole functions, cytokinesis and mitosis, and have been implicated in linking cell developmental processes with cell division and in cell differentiation [28]. Although the mechanism of action of Plks is still unclear, this may be because PLKs participate in regulation of the S-phase checkpoint, spindle assembly checkpoint and cytokinesis checkpoint, to ensure normal cell division [29]. The up-regulation of cell-related proteins in the p53 pathway suggests that µ-CTX GIIIB may inhibit the anti-tumor process of normal cells by promoting cell proliferation, and may have a potential carcinogenic effect.

In this study, although there was no difference in protein composition, cyclin genes such as *Ccna2, Ccnd1, Ccne1 and Ccne2* were up-regulated in transcriptome results. It also suggested that µ-CTX GIIIB affected normal cell cycle progression. In addition, we found two down-regulated genes related to tumors and apoptosis. The Gadd45a gene can be induced by a variety of DNA damage factors, and is also a downstream gene of the p53 protein, which is involved in cell cycle checkpoint control, DNA repair processes and signal transduction. Studies have shown that the deletion of *Gadd45a* significantly improves the abilities of cell adhesion, migration and invasion [30]. *Gadd45a*-deficient mice are more prone to tumor cell formation induced by DNA damage, and also show loss of genomic instability, oncogene transformation, centrosome amplification and cell senescence functions [31]. *Pmaip1* is a p53-inducing gene that can respond to DNA damage and has a pro-apoptotic function [32]. The down-regulation of *Gadd45a* and *Pmaip1* indicated that in addition to affecting the cell cycle, conotoxin may also cause DNA damage and induce tumor cell formation, leading to cell canceration.

Our study found that DEGs and DEPs in signal transduction-related pathways are enriched in the PI3K-Akt signaling pathway, MAPK signaling pathway and FoxO signaling pathway. Among them, breast cancer type 1 susceptibility protein (BRCA1), integrin alpha 3 (ITGA3), integrin beta 3 (ITGB3) and thousand and one amino acid protein kinase (TAO) were upregulated at both protein and gene levels. During the S/G(2) phase of the cell cycle, BRCA1 promotes DNA terminal excision and homologous recombination, which is essential for the selection of repair mechanisms for DNA double-strand breaks and for genomic integrity during cell division [33]. The effect of the toxin on the cell cycle may be mainly concentrated in the S phase and promote cell mitosis by affecting G1/S phase transition and S/G2 phase DNA synthesis. Both ITGA3 and ITGB3 belong to the integrin protein family, a class of cell surface adhesion molecules that can interact with extracellular matrix proteins. The expression of ITGA3 has been identified as a key molecular marker of various cancers in many omics studies [34, 35]. ITGB3 is involved in shaping the stromal and immune microenvironment, reprogramming tumor metabolism and maintaining tumor stem cells and other biological processes [36]. Integrin proteins mediate cell–cell and cell–extracellular matrix adhesion, and the up-regulation of related proteins may be due to the promotion of cell proliferation and migration by conotoxin. TAO contains more than 1,000 residues with a catalytic domain at the N-terminus that can activate the mitogen-activated protein (MAP) kinase cascade in vitro by phosphorylating MAP/ERK kinases 3 and 6 [37].

We also found some down-regulated proteins among the proteome data, but with no difference in the transcriptome data, including IκB kinase (IKK), protein phosphatase 3 catalytic subunit A (PPP3CA) and Bcl-2/E1B 19 kDa-interacting protein 3-like protein (Bnip3L). The IKK complex mediates phosphorylation of the inhibitory subunit of I kappaB alpha, leading to activation of nuclear factor kappaB (NF-κB), which plays a key role in inflammation, immunity, stress response, and apoptosis protection [38]. The down-regulation of IKK protein caused by CTX can directly affect activity of the NF-κB signaling pathway and inhibit the cellular inflammatory response. PPP3CA is the main catalytic subunit of PPP3 (protein phosphatase 3) in all skeletal muscle types and plays a role in gene expression of chronic muscle fibers, muscle tubule differentiation, muscle fiber growth and regeneration [39–42]. Imazu et al. found that transient expression of Bnip3L induced apoptosis of Rat-1 and HeLa cells, and it was one of the pro-apoptotic proteins containing BH3 [43]. Our results suggested that CTX can affect normal apoptosis of cells, maintain cell proliferation and protect cells from the conditions leading to cell death through apoptosis.

RAS proto-oncogene GTPase (RAS) and its direct downstream protein proto-oncogene serine/threonine-protein kinase (Raf) were up-regulated in the MAPK signaling pathway. There are three RAS proteins in mammalian cells, including the HRas proto-oncogene GTPase (HRas), the NRAS proto-oncogene GTPase (NRAS) and the KRAS proto-oncogene GTPase (KRAS), which are mainly involved in cell proliferation, survival, differentiation and other basic processes [44]. Dysregulation of the RAS pathway has been found in many cancers. The key role of RAS is to connect the activated receptor tyrosine kinase (RTKs) signal and the MAPK pathway in cells and to activate RAF to activate MEK1 and MEK2, which can induce the activity of a series of pro-growth factors [45]. Among signal transduction-related pathways, the enriched pathways and differentially-changed related proteins are mostly related to cell proliferation, apoptosis and differentiation, and most of them promote cell proliferation and inhibit normal cell apoptosis.

Other pathways related to DEPs and DEGs were mainly enriched after combined analysis included metabolic pathways, PPAR signaling pathway, biosynthesis of unsaturated fatty acids and fatty acid metabolism. The main DEPs included stearoyl-Coenzyme A desaturase 2 (SCD2), 3-hydroxy-3-methylglutaryl-coenzyme A synthase 1 (Hmgcs1), acyl-coenzyme A thioesterase (ACOT), perilipin (Plin), four and a half LIM domains protein (FHL) and cystathionine gamma-lyase (CSE). Except for HCH, their related genes and proteins showed the same variation trends.

The main role of SCD2 is to catalyze the synthesis of monounsaturated fatty acids (MUFAs), which is related to Parkinson’s disease and other central nervous system diseases [46, 47]. Recent studies have shown that Hmgcs1 has a potential carcinogenic effect in a wide range of human cancers and is highly expressed in most tumor types, reducing sensitivity to most drugs [48]. Perilipin-2 is a lipid droplet protein that wraps around a neutral lipid core and is involved in lipid metabolism in many cells, and has also been found to be associated with cancer progression [49]. The protein enrichment and differential expression of cancer-related proteins in lipid metabolism-related pathways indicate that µ-CTX GIIIB can affect lipid metabolism in normal cells, leading to various diseases and having potential carcinogenic effects. ACOT mainly plays a regulatory role in fatty acid metabolism and is considered as a key regulator of peroxisome lipid metabolism [50]. H2S derived from CSE plays an essential role in regulating various functions related to cardiovascular and cell growth, and exogenous H2S also has a certain regulatory effect on the expression of CSE in vascular endothelial cells under hypoxia [51]. Ying et al. found that the development of hypertension is closely related to the down-regulation of CSE expression [52]. In addition, some DEPs were also enriched in PPAR-α and PPAR-γ in the PPAR signaling pathway, indicating that the effect of µ-CTX GIIIB on cells could affect lipid metabolism of skeletal muscle cells, myocardial inflammatory response and myocardial injury, leading to myopathy.

## Conclusion

In summary, we identified 103 protein/gene pairs affected by µ-CTX GIIIB. The results show that µ-CTX GIIIB affects the normal progress of biological processes such as cell cycle regulation, DNA damage repair, lipid metabolism and anti-tumor effects. The enrichment of DEGs and DEPs in the signaling pathway is mainly related to cell cycle and signal transduction, suggesting that µ-CTX GIIIB may lead to carcinogenesis through its effects on cell cycle regulation, DNA damage repair, and activation of tumor factors, with potential carcinogenic effects. µ-CTX GIIIB, as a class of polypeptide toxoids, has very broad prospects for drug research. Our work provides further insight into the effects of toxins on biological processes inside cells and may facilitate wider use of µ-CTX GIIIB as a pharmacological tool.

### Supplementary Information

**Additional file 1: Table S1.** Description of DEGs; **Table S2.** Description of DEPs; **Table S3.** Related data of DEPs and DEGSs; **Table S4.** KEGG classification.

## Data Availability

The datasets are available from the corresponding author on request.
